# Predicting Suitable Habitat for *Sk^w^enk^w^ínem* (*Claytonia lanceolata*), a Culturally Significant Plant, Using a Reproducible Species Distribution Model

**DOI:** 10.1002/ece3.72615

**Published:** 2025-12-11

**Authors:** Hannah E. Pilat, David J. Ensing, Jason Pither

**Affiliations:** ^1^ Department of Biology University of British Columbia, Okanagan Campus Kelowna British Columbia Canada; ^2^ Summerland Research and Development Centre, Agriculture and Agri‐Food Canada Summerland British Columbia Canada

## Abstract

Colonialism and a changing climate have threatened culturally significant food plants and the well‐being of those who rely on them. An example is *sk* ^*w*^ *enk* ^*w*^ *ínem* (western spring beauty, *Claytonia lanceolata* : Pursh), which has declined in quality and accessibility for the *Secwépemc* People of Skeetchestn Indian Band. This study aims to provide predictions and visual tools to inform Skeetchestn's conservation efforts for their *sk* ^*w*^ *enk* ^*w*^ *ínem* patches, from a computationally reproducible species distribution model. Using a set of predictors informed by qualitative interviews with Skeetchestn community members (Informed model), and the 19 bioclimatic variables from WorldClim (WorldClim model), we predicted suitable habitat for *sk* ^*w*^ *enk* ^*w*^ *ínem* over its known range. For both our total study area and Skeetchestn Territory, we predicted a decrease in suitable habitat from the present to 2081–2100, based on the WorldClim CMIP6 climate change scenario SSP 5–8.5 (worst case). These predictions use Skeetchestn's knowledge of *sk* ^*w*^ *enk* ^*w*^ *ínem* and commonly used bioclimatic predictors to support Skeetchestn's goals of food sovereignty.

## Graphical abstract

*Sk* ^*w*^ *enk* ^*w*^ *ínem* ( *Claytonia lanceolata* ) is a culturally significant plant that has declined in quality and accessibility for the Secwépemc People of the Skeetchestn Indian Band. Using a reproducible species distribution model, this study provides predictions and visual tools to inform Skeetchestn's conservation efforts for their *sk* ^*w*^ *enk* ^*w*^ *ínem* patches.

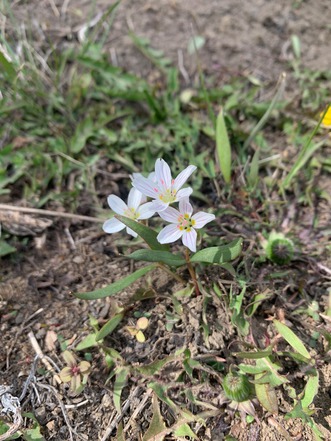

## 1 Introduction

Land‐use changes, including urbanization, recreational use, the introduction of invasive species, fire suppression, commercial logging, damming of waterways, and cattle grazing, threaten indigenous plants and the knowledge of those plants (Turner et al. 2000; Moore 2001; Turner and Turner 2008). A societal shift from the tending of plants to their exploitation, coupled with anthropogenic climate change, continues to cause declines in the abundance, quality, and accessibility of many plant species that are culturally important food and medicinal sources for Indigenous Peoples (Garibaldi and Turner 2004).

**FIGURE 1:**
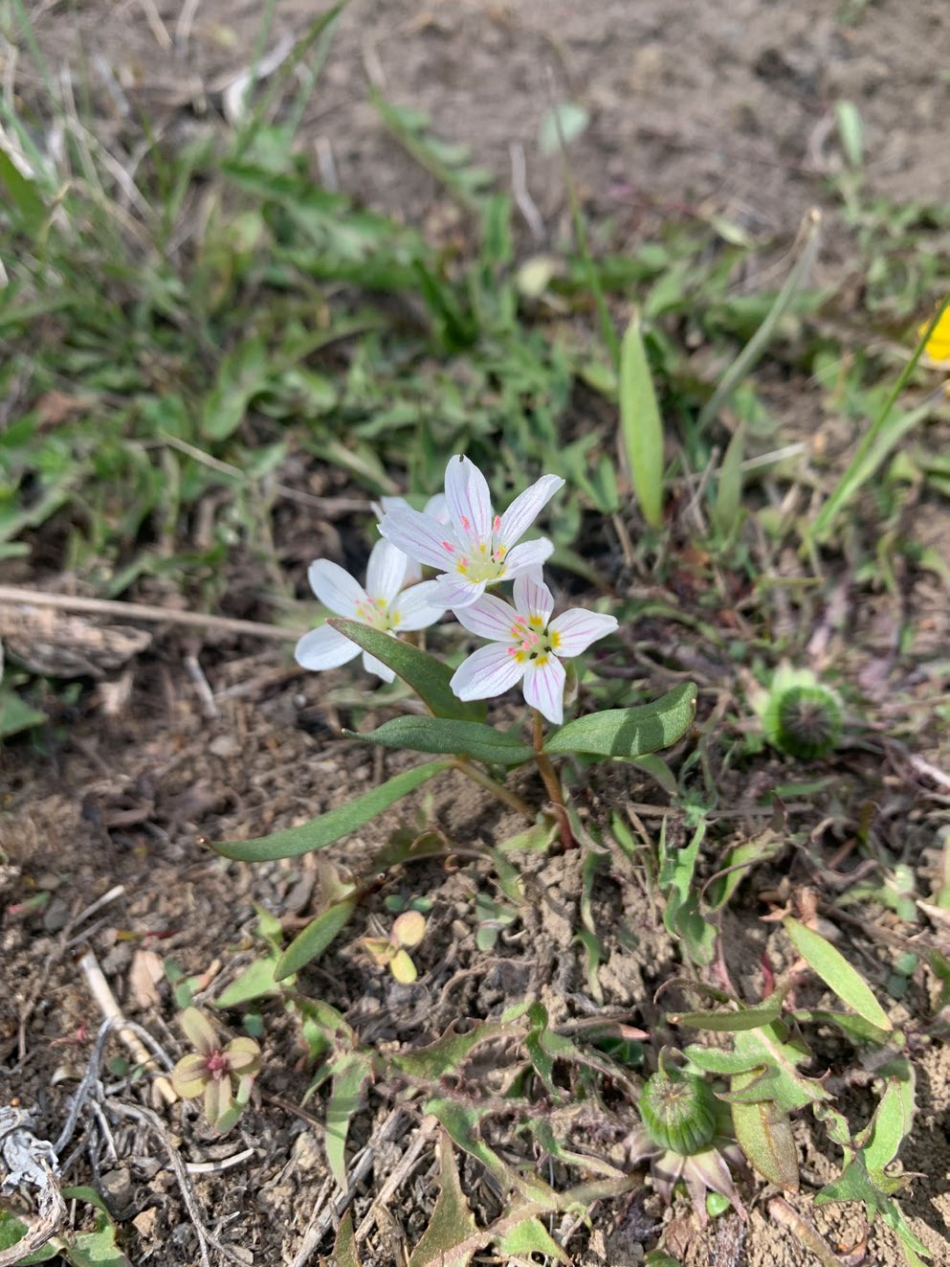
*Sk^w^enk^w^ínem* ( *Claytonia lanceolata* ) plant in bloom.

*Sk* ^*w*^ *enk* ^*w*^ *ínem* ( *Claytonia lanceolata* ; Montiaceae, western spring beauty, mountain potato; Figure 1) is a food plant that is culturally significant to the *Secwépemc* People and is declining in quality and abundance (Turner 2014, Lizzy Ignace, personal communication February 2022). *Sk* ^*w*^ *enk* ^*w*^ *ínem* is a geophyte that produces corms, which are harvested and consumed by Indigenous Peoples across what is now known as North America. *Sk* ^*w*^ *enk* ^*w*^ *ínem* corms were once a significant portion of the *Secwépemc* diet (Turner 1997, 2014; Ignace et al. 2016). However, soil compaction from cattle grazing may be limiting high‐quality habitat for *sk* ^*w*^ *enk* ^*w*^ *ínem*, constraining the size of corms produced by the plant and therefore its accessibility as a meaningful food source (Turner 1997, 2014, *Secwépemc* Elder Mary Thomas in Turner et al. 2000; Pilat 2024). A reduction in Indigenous care‐taking practices has been identified as a potential cause for the reduction in the abundance and the quality of *sk* ^*w*^ *enk* ^*w*^ *ínem* corms and a decline in *sk* ^*w*^ *enk* ^*w*^ *ínem* patch productivity (Turner 2014, Elder Mary Thomas in Ignace et al. 2016).

Ongoing changes in climate compound the challenges presented by a reduction in caretaking practices. *Sk* ^*w*^ *enk* ^*w*^ *ínem* shoots emerge from the soil when the plant is less likely to be negatively impacted by freezing, frost, and drought (Briceño et al. 2015; Gezon et al. 2016). Sufficient snowpack insulates *sk* ^*w*^ *enk* ^*w*^ *ínem*'s vegetative and reproductive organs at or just above 0°C during winter, leaving them dormant and viable for the growing season (Miller and Chambers 2006; Briceño et al. 2015). Prior to snowmelt, air temperatures are much cooler than the soil, so the phenology of *sk* ^*w*^ *enk* ^*w*^ *ínem* is an adaptation that protects the plant from adverse weather conditions (Briceño et al. 2015). Since *sk* ^*w*^ *enk* ^*w*^ *ínem* emerges immediately following snowmelt, it is particularly sensitive to snowpack and the timing of snowmelt (Gezon et al. 2016). The timing of snowmelt is advancing due to climate change, placing *sk* ^*w*^ *enk* ^*w*^ *ínem* plants at risk of frost damage (Dunne et al. 2003; Gezon et al. 2016).

Species distribution models (SDMs) are correlative statistical frameworks that fit a species' occurrence data to environmental predictor variables to describe species' relationships to their environment (Guisan and Zimmermann 2000; Elith et al. 2006; Elith and Leathwick 2009; Yates et al. 2018; Wilkinson et al. 2019; Araújo et al. 2019; König et al. 2021). SDMs are the most widely employed frameworks for predicting the impacts of climate change on biodiversity because they generate occurrence probabilities, habitat suitability indicators, and response curves for the species‐environment relationship (Guisan et al. 2013; Ehrlén and Morris 2015; Ferrier 2016; Liu et al. 2019; Zurell et al. 2020). Understanding the potential effects of climate change on the suitability of habitat for culturally significant plant species is key to understanding the future accessibility of these plants for the people who rely on them.

The modeling of culturally significant species' distributions is not rare in the SDM literature. However, few studies acknowledge the cultural significance of their focal organisms, or the Territories in which those studies were conducted. A handful of studies (Baumflek et al. 2015; Doyle‐Yamaguchi 2021; Campbell et al. 2022; Mucioki et al. 2022; Koutzoukis et al. 2024) describe work with Indigenous Peoples to produce SDMs, and these studies served as examples for our work. To the best of our knowledge, there are no computationally reproducible SDM studies informed by Indigenous Knowledge. With this study, we aim to start filling this notable research gap. This SDM study complements a qualitative study with Skeetchestn community members, investigating the biology of *sk* ^*w*^ *enk* ^*w*^ *ínem* and its importance to the Skeetchestn community (Pilat 2024). Both qualitative and quantitative studies tell a more complete story of *sk* ^*w*^ *enk* ^*w*^ *ínem* under the framework of Two‐Eyed Seeing, while also contributing independently to the documented knowledge of *sk* ^*w*^ *enk* ^*w*^ *ínem*. Two‐Eyed Seeing, as described by Albert and Murdena Marshall, is the *Mi'kmaw* concept of using two different knowledge systems for a broader perspective (Bartlett et al. 2012). Similarly, Skeetchestn community member Ron Ignace describes a comparable concept called “Walking on Two Legs” (Dickson‐Hoyle et al. 2022). The broader project, of which this study forms a part, is demonstrative of a Two‐Eyed Seeing framework to better understand the ecology of *sk* ^*w*^ *enk* ^*w*^ *ínem*. We use an SDM informed by Skeetchestn Knowledge and the same model algorithm using common bioclimatic predictors to compare predictions and gain a better understanding of *sk* ^*w*^ *enk* ^*w*^ *ínem*'s spatial ecology.

Our objective is to provide a reproducible example of a species distribution model that is focused on a culturally significant plant species, *sk* ^*w*^ *enk* ^*w*^ *ínem*, and in doing so, address the following research questions: (1) What is the predicted distribution of suitable habitat for *sk* ^*w*^ *enk* ^*w*^ *ínem* in the present and from 2081 to 2100, under the most extreme climate change scenario, CMIP6 SSP 5–8.5, (2) What is the effect of projected climate change scenarios on the available habitat for *sk* ^*w*^ *enk* ^*w*^ *ínem* in our study extent and within Skeetchestn Territory? The goal in answering these questions is to guide food sovereignty efforts in Skeetchestn Territory by determining suitable habitat for *sk* ^*w*^ *enk* ^*w*^ *ínem* now and with predicted climate change.

### 1.1 Author Positionality

Our research team is composed of an Indigenous researcher (HP) who is Woodland Cree from Treaty 6 Territory with *Michif* and mixed settler ancestry, and settler scholars (DE, JP). In 2021, DE, a vegetation ecologist with Agriculture and Agri‐Food Canada, partnered with Skeetchestn Natural Resources Corporation (SNRC), with whom the project connects to the broader Skeetchestn community, on a project to revitalize Skeetchestn's agro‐ecology. The *Revitalizing Sk* ^*w*^ *enk* ^*w*^ *ínem Agro‐Ecology* project began at the invitation of SNRC to investigate the Skeetchestn community's depleting food resources. SNRC employees and Skeetchestn community members have been involved in the research design, fieldwork, community events, and were the foundation of this research. JP is an ecologist who joined the project in a supervisory capacity for HP in 2022 and is continuing his relationship with the community with in‐kind consultations on improving their capacity for data management and data sovereignty. We humbly acknowledge this study would not exist without the Skeetchestn community welcoming us to their Territory and sharing their Knowledge with us.

## 2 Materials and Methods

SDM workflows require the analyst to make many decisions, and in the absence of full transparency and pre‐registration, this can introduce bias (e.g., favoring decisions that yield more compelling outcomes). We therefore pre‐registered our analysis plan and used the ODMAP (Overview, Data, Model, Assessment and Prediction) reporting protocol (Zurell et al. 2020) to report the model workflow (see Data Availability Statement). The only deviation from our pre‐registration is that we changed the name “Bioclim model” to “WorldClim model” for consistency with the dataset used. We first tested the SDM workflow with the modeling choices reported in our ODMAP protocol using *Ranunculus glaberrimus* (Ranunculaceae, sagebrush buttercup) occurrence records as an independent (i.e., “surrogate”) test species. *R. glaberrimus* often co‐occurs with *sk* ^*w*^ *enk* ^*w*^ *ínem*, but has no known cultural significance (Pilat 2024). It therefore served as an ideal dataset with which to learn the specifics of the tidysdm procedures (see below) and to test our proposed workflow, as specified in the ODMAP protocol. Providing a complete and transparent description of our analysis protocol (ODMAP), combined with using an independent dataset ( *R. glaberrimus* ) to develop and test our protocol, simultaneously maximizes the computational reproducibility of our models and their reliability by minimizing potential bias during the model training phase.

We produced continuous habitat suitability indices and binary suitable habitat maps for: (1) the occupied niche of *sk* ^*w*^ *enk* ^*w*^ *ínem*, from the West Coast of North America to the Great Continental Divide, as evidenced in Global Biodiversity Information Facility (GBIF) occurrence records, and (2) Skeetchestn Traditional Territory. The continuous habitat suitability and binary suitable habitat maps are products of realized and potential species distribution models. The realized species distribution model for *sk* ^*w*^ *enk* ^*w*^ *ínem* is predicted over its entire known range in the present time, with both Informed predictors and climate‐related predictors as input into separate models. The Informed predictors were selected based on the results of a qualitative study conducted with Skeetchestn community members (Pilat 2024). The Informed predictors consist of soil temperature and pH, elevation, landcover, and anthropogenic biomes. The climate‐related predictors are a set of 19 bioclimatic variables with temperature and precipitation values from 1970 to 2000, all sourced from the WorldClim website (See Data Availability Statement). The potential species distribution model for *sk* ^*w*^ *enk* ^*w*^ *ínem* is predicted over its entire range in the future, under predicted climate change scenario SSP 5–8.5 from the CMIP6 model, with WorldClim predictors from 2081 to 2100.

Following the predictions over *sk* ^*w*^ *enk* ^*w*^ *ínem*'s entire range, we cropped the extent to Skeetchestn Traditional Territory to produce maps for Skeetchestn's use. These maps of Skeetchestn are not publicly available and are stewarded by Skeetchestn Natural Resources Corporation, following the principles of Ownership, Control, Access, and Possession (OCAP; First Nations Information Governance Centre 2024). These maps may be used to inform future conservation and revitalization efforts within Skeetchestn Territory by mapping suitable habitat now and where we can expect suitable habitat to exist with a changing climate.

### 2.1 Study Areas

**FIGURE 2:**
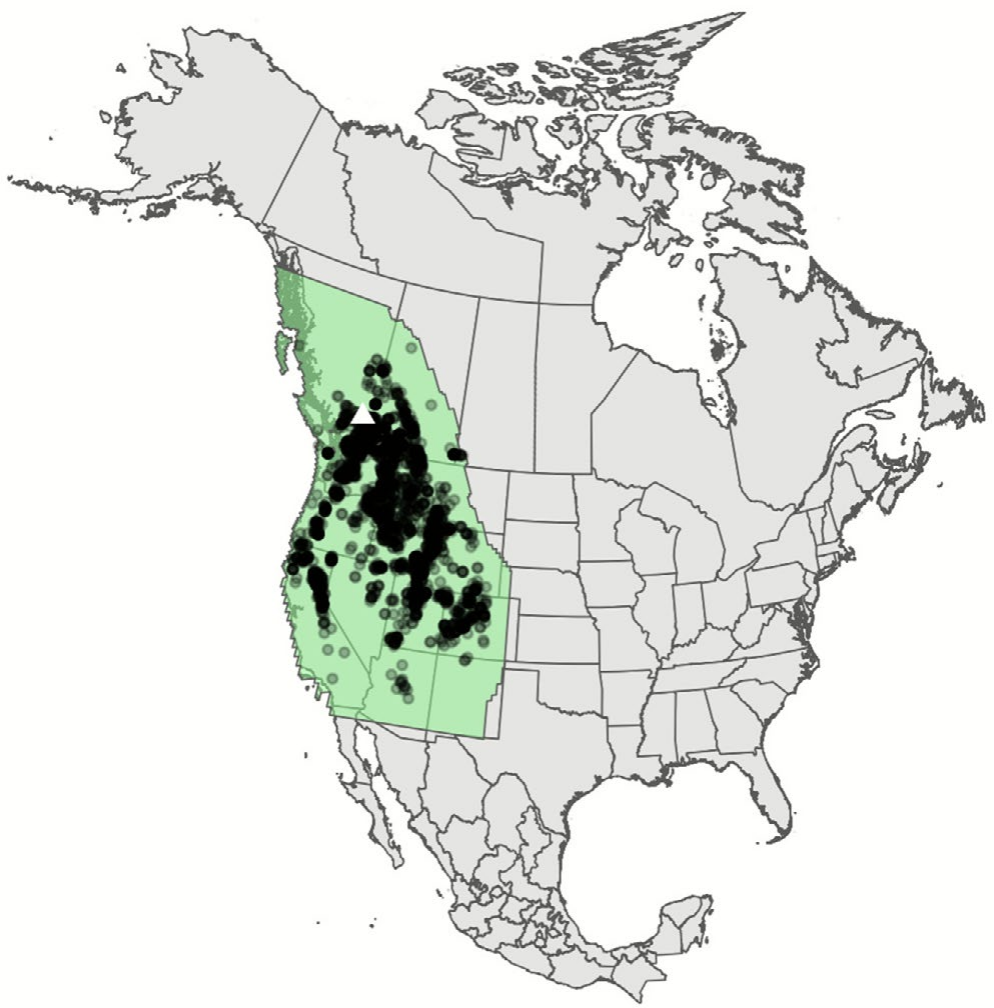
Boundary of our study extent (green polygon) with *sk^w^enk^w^ínem* ( *Claytonia lanceolata* ) occurrence points, within the context of North America. Map projection used is the North America Albers Equal Area conic projection. The white triangle indicates the general location of Skeetchestn Territory.

We constructed all SDMs using a study extent that encompasses the current, known distribution of *sk* ^*w*^ *enk* ^*w*^ *ínem*, which is approximately 4,023,545 km^2^ (Figure 2). We chose this extent to capture the full fundamental niche of the species by encompassing all known occurrence records and their full span of associated environmental conditions (Elith and Leathwick 2009; Thuiller et al. 2009; Gogol‐Prokurat 2011; Essl et al. 2024). To accommodate potential shifts in distribution with climate change, our full study extent also includes a buffer beyond the extent of occurrence records: a 15° buffer to the north and a 2° buffer to the east. We cropped the model results to Skeetchestn Territory, which is approximately 7190 km^2^.

### 2.2 Workflow

**FIGURE 3:**
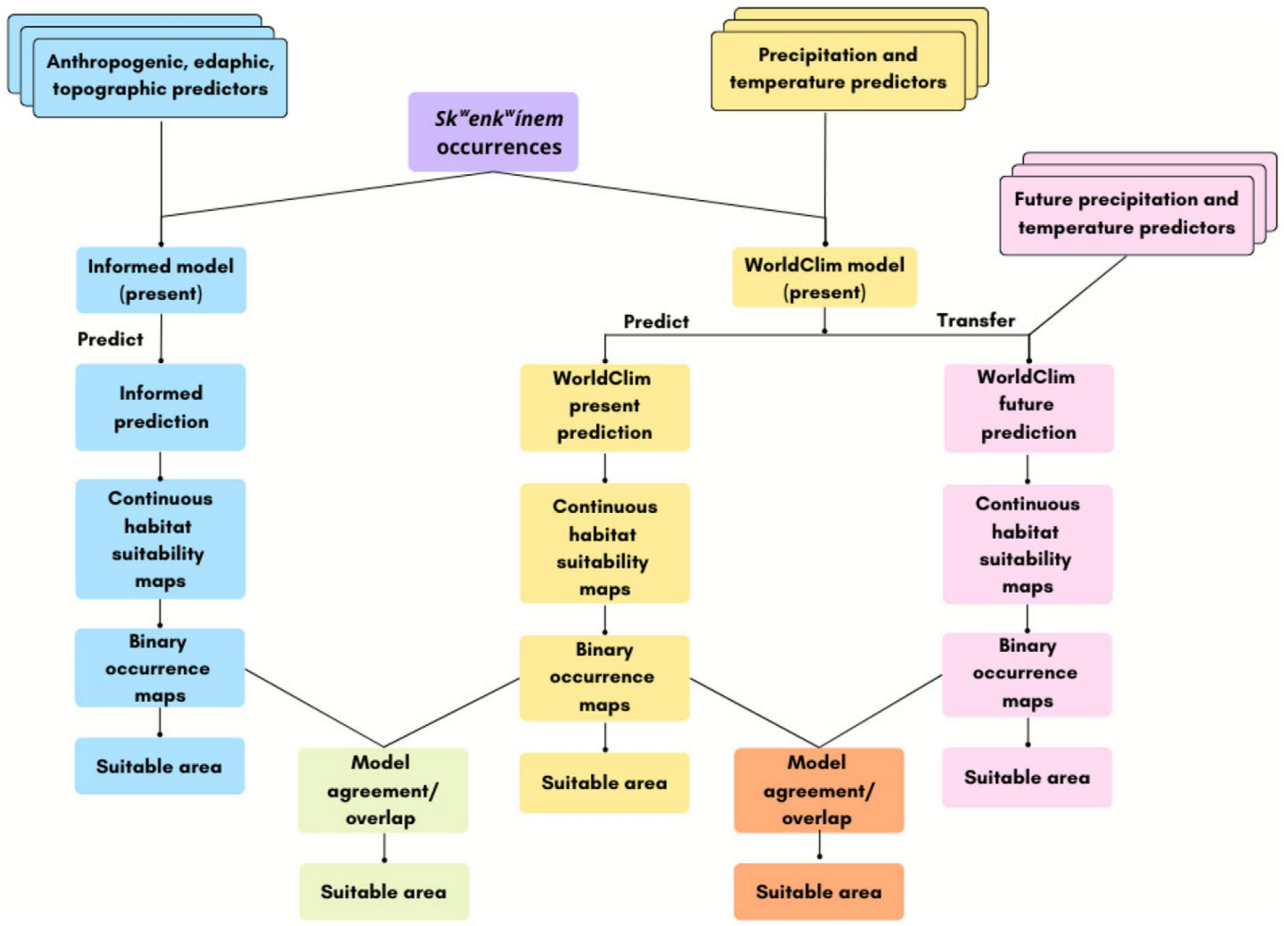
*Sk^w^enk^w^ínem* ( *Claytonia lanceolata* ) species distribution model workflow with both Informed and WorldClim models.

Our overall workflow generated species distribution models using the same occurrence records with WorldClim data (referred to as the “WorldClim model”; Figure 3), and data informed from qualitative interviews and analysis with Skeetchestn community members (Pilat 2024) as predictors (referred to as the “Informed model”; Figure 3). Both the WorldClim and Informed predictor datasets were formatted as rasters. We transferred the WorldClim model to make a future habitat suitability prediction from 2081 to 2100 under the CMIP6 climate change scenario SSP 5–8.5, referred to as the WorldClim future prediction (Figure 3). Both models use publicly available data and the same model settings. Choices made during the modeling process are included in detail in the ODMAP Protocol (see Data Availability Statement). Analysis was completed in R version 4.3.2 (R Core Team 2023) using tidysdm version 0.9.3 (Leonardi et al. 2024). A detailed list of packages and their versions is included in the ODMAP Protocol.

### 2.3 Occurrence Data

We downloaded occurrence records from the Global Biodiversity Information Facility (GBIF.org 2024) through the rgbif package (Chamberlain et al. 2024). We selected records from human observation and preserved specimens, with coordinates available, and filtered out erroneous records with 0.0 as the coordinates. We included preserved specimens to have as many occurrences as possible to best represent the species' realized niche, past and present (Smith et al. 2023). Using more occurrence records results in a more complex model, which tends to produce better predictions of suitable habitat (Liu et al. 2019; Brun et al. 2020). We selected records between 1940 and 2024 to encompass the timespan of the present‐day climate data, from 1970 to 2000, and to minimize mismatches between climate and both historical and recently established *sk* ^*w*^ *enk* ^*w*^ *ínem* populations.

Subsampling of the occurrence dataset through spatial thinning of the records reduces spatial bias in human observations (Baker et al. 2022; Barber et al. 2022). We began with 3541 occurrences and thinned them in a two‐step process, following the tidysdm workflow. The first thinning step limited occurrences to one per cell of the multilayer raster with predictor layers used in the model, which retained 2565 occurrences. The second thinning step constrained occurrences to a minimum intervening distance of 15 km, which retained 859 occurrences.

To generate pseudoabsences, we sampled 10 times the number of presences so reproduction with differing numbers of occurrences will maintain the same relative proportion of occurrences versus pseudoabsences. We generated pseudoabsences through radius‐restricted background sampling, which has been shown to improve predictions made by the model Maximum Entropy (MaxEnt; Phillips et al. 2006, 2009; Anderson and Raza 2010; Barve et al. 2011; Acevedo et al. 2012; Barber et al. 2022). Using the radius‐restricted sampling method introduces similar spatial biases as the occurrence data have from opportunistic community science collection. The effect of sampling bias in our occurrence data is therefore reduced by introducing the same bias in our pseudoabsence sampling approach (Phillips et al. 2009; Barber et al. 2022). Pseudoabsence samples were taken from a buffer around occurrence points, and sampled randomly within those buffers, without replacement. The circular buffer (a “disc”) is within a minimum of 50 km and a maximum of 75 km around occurrences, through the dist_disc argument. During the *Ranunculus* training phase, we found pseudoabsence sampling discs close to the occurrences (minimum distance 5–20 km away) to result in poor discrimination of presences from pseudoabsences, and therefore poor model performance.

### 2.4 Predictor Data

We used the 19 bioclimatic variables from WorldClim in the WorldClim model. The WorldClim data provides annual, quarterly, minimum, and maximum temperature and precipitation measurements for the present day, as well as future predictions created through the CMIP6 models (Eyring et al. 2016). The CMIP6 models account for greenhouse gas emissions scenarios combined with scenarios of varying degrees of climate warming reduction policies implemented. We selected the SSP 5–8.5 HadGEM3‐GC31‐LL model for our analysis (Eyring et al. 2016; Roberts 2017; Met Office Hadley Centre 2024). For our current distribution predictions, we used the 19 bioclimatic variables from 1971 to 2000. The future projections used the worst‐case Shared Socioeconomic Pathways model (SSP 5–8.5) from CMIP6 for the years 2081–2100, the most advanced temporal predictions, to illustrate the most pessimistic future climate change scenario. We used 30 arcsecond resolution data for both present and future predictions, the highest resolution available from WorldClim.

The WorldClim predictors are available at 30 arcsecond resolution (1 km by 1 km at the equator) and referenced using the World Geodetic System 1984 (WGS1984). We transformed the Informed rasters to WGS1984, cropped to our study extent, and resampled to all have matching resolutions of 30 arcseconds to allow for direct comparison between the Informed and WorldClim present‐day models. We resampled the numeric rasters using bilinear interpolation, and categorical rasters using the nearest neighbor method. We prepared the predictor rasters from both datasets for modeling using the terra (Hijmans 2023) and sf (Pebesma et al. 2023) packages, which interface well with tidysdm.

### 2.5 Predictor Selection

Predictors are typically selected for their ecological and statistical relevance in predicting species' distributions (Guisan and Zimmermann 2000; Adde et al. 2023). We used two sets of predictor variables for our two models to assess model performance between climate‐only variables (WorldClim model) and variables tailored to our study system (Informed model). Our selection of variables for the Informed model is a combination of variables informed through the qualitative analysis with Skeetchestn community members (Pilat 2024) and a literature review (Table 1). We selected datasets with the highest resolution available for our models to better represent the high climatic heterogeneity of our study area (Essl et al. 2024). For each model, we selected a subset of uncorrelated predictors by removing variables with Pearson's *r* > 0.8. Specifically, the tidysdm package created a pairwise matrix of the predictors and of those that are highly correlated; the predictor with the largest mean absolute correlation value was suggested for removal from analysis (Leonardi et al., n.d.). The process of selecting expert‐chosen predictors and filtering out highly correlated predictors balances the need for biologically significant predictors that are not correlated to avoid overrepresenting the influences of similar variables in the predictions (Adde et al. 2023).

**TABLE 1:** Predictor variables used in the informed species distribution model for *sk* ^*w*^ *enk* ^*w*^ *ínem* ( *Claytonia lanceolata* ).

Variable name	Description/justification	Source
Anthropogenic biomes^a^	Anthropogenic biomes account for ecological biomes that have been greatly influenced by humans. Examples include urban areas, rural settlements, rangeland, different types of cropland, etc., which are thought to be limiting suitable habitat for *sk* ^*w*^ *enk* ^*w*^ *ínem* (Pilat 2024)	Ellis and Ramankutty (2008)
Elevation^a^	*Sk* ^*w*^ *enk* ^*w*^ *ínem* is known to grow between sea level and 3600 m in elevation (Stewart and Wiens 1971), but is localized to montane habitats in Skeetchestn Territory (Pilat 2024)	Commission for Environmental Cooperation (2023)
Landcover^a^	*Sk* ^*w*^ *enk* ^*w*^ *ínem* is known to grow in meadows in Douglas fir forests (Pilat 2024). Landcover is therefore important to include as a predictor, to classify different habitats, such as meadows	Commission for Environmental Cooperation (2020)
Soil pH, 0–5 cm	Soil pH in H_2_O X 10, 0–5 cm below the surface. Soil pH was found to be an important predictor for plants growing in meadows in the Swiss Alps (Buri et al. 2020) and for influencing alpine plant community composition (Vonlanthen et al. 2006)	International Soil Reference and Information Centre (ISRIC) (2022)
Soil temperature seasonality (°C), 0–5 cm^a^	SBIO4–temperature seasonality (standard deviation × 100), 0–5 cm below the surface. Temperature seasonality is the variability in soil temperature. This is important to consider for *sk* ^*w*^ *enk* ^*w*^ *ínem*, which requires soil to remain above 0°C over winter (Miller and Chambers 2006; Briceño et al. 2015)	van den Hoogen et al. (2021)

*Note:* Variables were chosen based on the interviews and qualitative analysis (Pilat 2024).

^a^ Indicates uncorrelated variables included in our models.

Plant species distributions are not solely governed by climatic conditions; soil characteristics are also determinants of plant distributions in space and are important for determining habitat suitability, especially on a continental scale (Ni and Vellend 2022). We included soil temperature and pH as predictors, 0–5 cm below the surface for each, which is a relevant depth for *sk* ^*w*^ *enk* ^*w*^ *ínem*, whose corms are known to grow at shallow depths (Pilat 2024). Similarly, landcover, through habitat fragmentation and anthropogenic pressures, is an important driver of plant species' distributions (Chauvier et al. 2021). Through prior interviews and qualitative analysis, we learned *sk* ^*w*^ *enk* ^*w*^ *ínem* is commonly found in forest openings, ranging from large meadows to small pockets where the forest canopy opens (Pilat 2024), making landcover an important predictor to include in our model. To add a layer of human influence, we included a predictor layer of anthropogenic biomes. Anthropogenic biomes are ecosystem types with significant influence by humans, such as urban areas, settlements, cropland, and rangeland (Ellis and Ramankutty 2008). Including the human influence on ecosystems is relevant for our study, as cattle grazing is believed to have a large influence on *sk* ^*w*^ *enk* ^*w*^ *ínem* health, and potentially its distribution in space (Elder Mary Thomas in Turner et al. 2000; Ignace et al. 2016; Thomas et al. 2016; Pilat 2024).

### 2.6 Contribution of Predictor Variables

We determined the contribution of variables visually by marginal response curves, which depict the contribution of one variable at a time by setting the other variables to their mean. For a quantitative comparison, we compared variable importance. Variables with high importance (mean dropout loss closer to 1) have the greatest influence on the model's predictive performance when they are independently removed from the model (Naimi and Araújo 2016; Zurell 2020). Variables with low importance (mean dropout loss closer to 0) have little influence on the model's predictive performance when removed (Naimi and Araújo 2016; Zurell 2020).

### 2.7 Model Evaluation and Selection

We evaluated SDMs using cross‐validation, which tests the model's ability to discriminate presences from pseudoabsences (Elith et al. 2006; Thuiller et al. 2009; Barbet‐Massin et al. 2012; Yates et al. 2018; Hao et al. 2019). We used *k* = 5 cross‐validation folds, where the data were partitioned into 5 groups and assigned to spatial blocks. One of these 5 groups (20% of the data) is withheld from model training and reserved for model evaluation (Naimi and Araújo 2016; Roberts et al. 2017; Soley‐Guardia et al. 2024).

The tidysdm package allows for the use of multiple model algorithms: we selected Maximum Entropy (MaxEnt) and random forest (rf) for their ability to use categorical predictors and for their high predictive capabilities as species distribution models (Valavi et al. 2022). We initially also used a generalized linear model (GLM) and a generalized boosted model (GBM), but neither could accommodate the many levels in each of the categorical predictors in the Informed model. We assessed model predictive performance using the area under the receiver operating characteristic curve (AUC), which assesses a model's ability to discriminate between presences and absences (Valavi et al. 2022). Models performing at 0.5 AUC discriminate presences and absences no better than random, and 1.0 AUC is considered perfect discrimination (Skroblin et al. 2021; Valavi et al. 2022). An AUC below 0.7 is considered poor, between 0.7 and 0.8 considered acceptable, between 0.8 and 0.9 considered excellent, and 0.9 or greater considered outstanding performance (Elith et al. 2006; Gogol‐Prokurat 2011; Ensing et al. 2013).

### 2.8 Ensemble Approach

We used an ensemble approach to account for uncertainties in the individual algorithms used to classify habitat suitability (Araújo and New 2007; Hao et al. 2019; Araújo et al. 2019; Thuiller et al. 2019). Ensemble models average the individual predictions made from single species distribution models using individual algorithms for combined predictions (Thuiller et al. 2009; Hao et al. 2019; Liu et al. 2019). MaxEnt and random forest were each run with 10 combinations of tuned hyperparameters, with the best performing combinations selected for inclusion in the ensemble by tidysdm. With the tidysdm pipeline, only the best performing models in the ensemble (AUC > 0.8) were retained and contributed to the predictions. The resultant AUC values in the ensemble were the mean AUC values for each model.

### 2.9 Classification of Suitable or Unsuitable Habitat

We produced continuous habitat suitability indices, ranging from 0 (unsuitable) to 1.0 (perfectly suitable), and binary predictions of suitable or unsuitable habitat. The binary predictions result from selecting a threshold in which cells are classified as suitable (above the threshold) or unsuitable habitat (below the threshold). We used the optimized true skill statistic (maxTSS) as the threshold for suitable versus unsuitable habitat. We classified the raster cells of the binary predictions as “suitable”, “unsuitable”, and “overlap”. The overlap consists of raster cells classified as suitable by both models in each prediction. To calculate the area of suitable habitat, we projected our habitat suitability rasters to the North America Albers Equal Area conic projection (ESRI:102008), then multiplied the number of cells in each class by the individual cell area.

## 3 Results

### 3.1 Model Performance

MaxEnt and random forest produced excellent model performances for both the WorldClim and Informed models. The WorldClim model produced outstanding model performances with both MaxEnt and random forest algorithms (AUC > 0.9; Table 2). MaxEnt and random forest in the Informed model produced excellent predictive performance (AUC > 0.85), although with a lower AUC than the WorldClim model (Table 2). Both MaxEnt and random forest in the WorldClim and Informed models performed above our threshold for inclusion in the predictions (AUC > 0.8; Table 2).

**TABLE 2:** Strong species distribution model performance (area under receiver operating characteristic curve [AUC]) for informed and WorldClim models of *sk* ^*w*^ *enk* ^*w*^ *ínem* ( *Claytonia lanceolata* ) in western North America.

Algorithm	Informed model (mean AUC ± SEM)	WorldClim model (mean AUC ± SEM)
MaxEnt	0.88 ± 0.014	0.91 ± 0.018
Random forest (rf)	0.87 ± 0.014	0.92 ± 0.013

*Note:* Values are mean AUC for each model, with standard error (SEM), after combining into an ensemble. AUC and SEM values are rounded to 2 and 3 decimal places, respectively. Higher AUC values indicate higher predictive performance.

### 3.2 Predictor Variable Selection and Contributions

**FIGURE 4:**
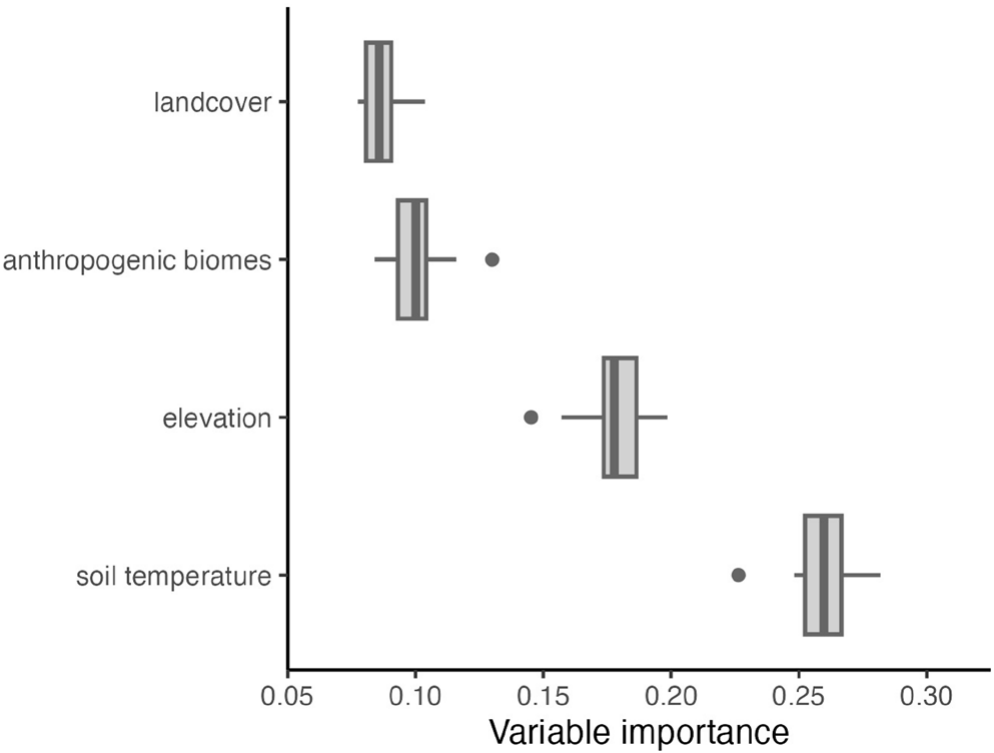
Predictor importance for the Informed species distribution model of *sk^w^enk^w^ínem* ( *Claytonia lanceolata* ) in western North America. The variable importance is mean dropout loss (one minus AUC loss after permutations) for the model predictor variables, with higher values indicating more influential variables on the model performance and predicting habitat suitability. Elevation is in meters, and soil temperature is soil temperature seasonality (standard deviation). On each box, the box edges represent the 25th and 75th percentiles, the central line indicates the median, the points indicate outliers, and the whiskers extend to the most extreme data points not considered outliers.

**FIGURE 5:**
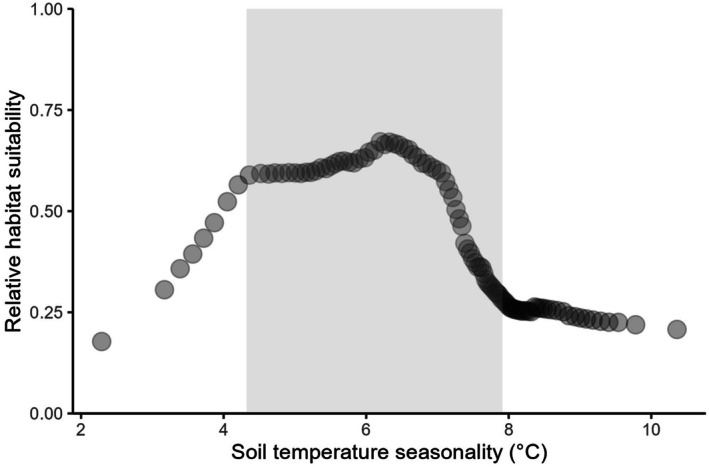
Marginal response curve, based on the Informed species distribution model of present conditions, between soil temperature seasonality (standard deviation), 0–5 cm below the surface (°C), and predicted habitat suitability of *sk^w^enk^w^ínem* ( *Claytonia lanceolata* ). Relative habitat suitability for *sk^w^enk^w^ínem* ranges from 0 = unsuitable to 1 = most suitable. Relative habitat suitability was predicted for soil temperature seasonality while other variables in the Informed model were held constant at their mean relative habitat suitability. The shaded area represents values within Skeetchestn Territory.

**FIGURE 6:**
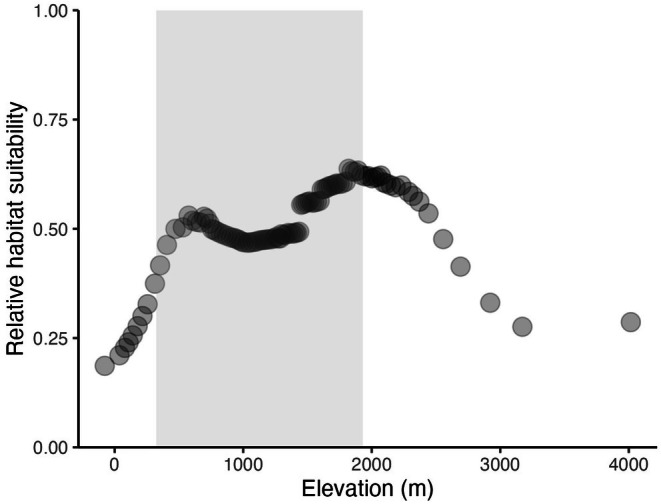
Marginal response curve, based on the Informed species distribution model of present conditions, between elevation (m) and predicted habitat suitability of *sk^w^enk^w^ínem* ( *Claytonia lanceolata* ). *Sk^w^enk^w^ínem* habitat suitability is predicted to increase with increasing elevation, up to 2000 m. Relative habitat suitability for *sk^w^enk^w^ínem* ranges from 0 = unsuitable to 1 = most suitable. Relative habitat suitability was predicted for elevation while other variables in the Informed model were held constant at their mean relative habitat suitability. The shaded area represents values within Skeetchestn Territory.

Uncorrelated predictor variables are shown in Tables 1 and 3. The two most influential predictors in the Informed model were soil temperature seasonality (0–5 cm below the surface) and elevation (m), which did not differ greatly in importance (Figure 4). Relative habitat suitability for *sk* ^*w*^ *enk* ^*w*^ *ínem* generally decreased with increasing soil temperature seasonality 0–5 cm below the surface, with maximum habitat suitability at around a 6.5°C standard deviation in soil temperature (Figure 5). Relative habitat suitability for *sk* ^*w*^ *enk* ^*w*^ *ínem* generally increased with increasing elevation, with the highest relative habitat suitability around 2000 m (Figure 6).

**TABLE 3:** Predictor variables used in the WorldClim species distribution model for *sk* ^*w*^ *enk* ^*w*^ *ínem* ( *Claytonia lanceolata* ).

Variable abbreviation	Variable full name/description
BIO1	Annual mean temperature
BIO2^a^	Mean diurnal range (mean of monthly [max temp–min temp])
BIO3^a^	Isothermality (BIO2/BIO7) (×100)
BIO4	Temperature seasonality (standard deviation ×100)
BIO5	Max temperature of warmest month
BIO6	Min temperature of coldest month
BIO7^a^	Temperature annual range (BIO5–BIO6)
BIO8^a^	Mean temperature of wettest quarter
BIO9^a^	Mean temperature of driest quarter
BIO10	Mean temperature of warmest quarter
BIO11	Mean temperature of coldest quarter
BIO12	Annual precipitation
BIO13^a^	Precipitation of wettest month
BIO14	Precipitation of driest month
BIO15^a^	Precipitation seasonality (coefficient of variation)
BIO16	Precipitation of wettest quarter
BIO17	Precipitation of driest quarter
BIO18^a^	Precipitation of warmest quarter
BIO19	Precipitation of coldest quarter

^a^ Indicates uncorrelated variables included in our model.

**FIGURE 7:**
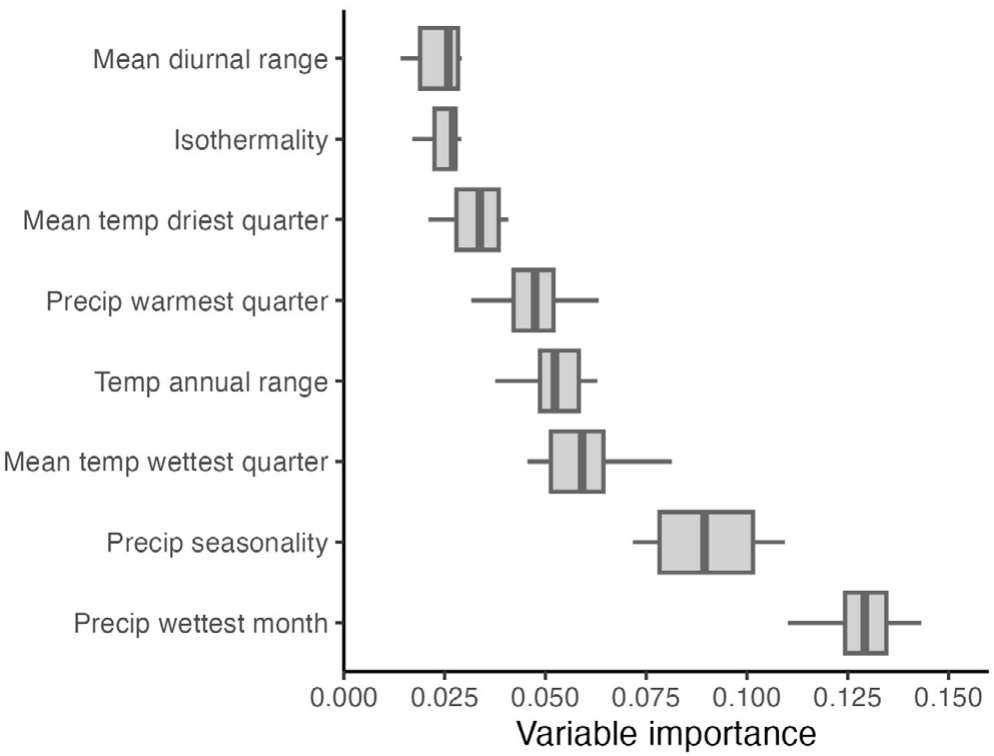
Predictor importance for the WorldClim species distribution model of *sk^w^enk^w^ínem* ( *Claytonia lanceolata* ) in western North America. The variable importance is mean dropout loss (one minus AUC loss after permutations) for the model predictor variables, with higher values indicating more influential variables on the model performance and on predicting habitat suitability. Predictors include, in order from least to most important: Annual mean diurnal range (°C), isothermality (%), mean temperature of the driest quarter (°C), precipitation of the warmest quarter (mm), annual temperature range (°C), mean temperature of the wettest quarter (°C), precipitation seasonality (%), and precipitation of the wettest month (mm). On each box, the box edges represent the 25th and 75th percentiles, the central line indicates the median, the points indicate outliers, and the whiskers extend to the most extreme data points not considered outliers.

**FIGURE 8:**
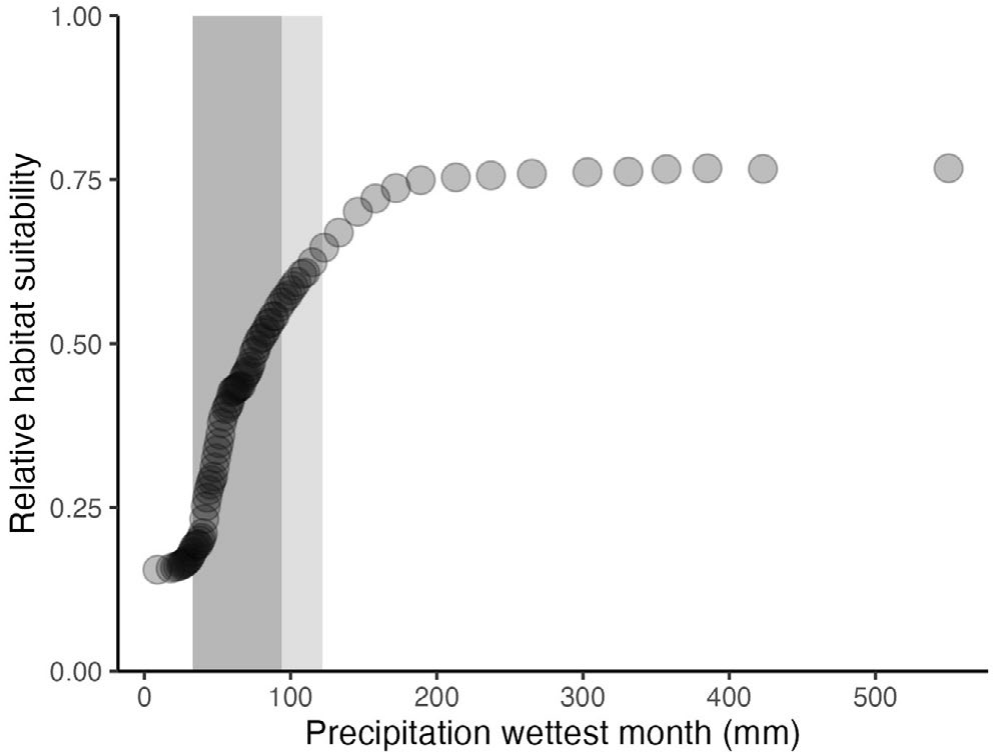
Marginal response curve, based on the WorldClim species distribution model of present conditions, indicating *sk^w^enk^w^ínem* ( *Claytonia lanceolata* ) habitat suitability is predicted to increase with precipitation of the wettest month (mm, Bio13). Relative habitat suitability for *sk^w^enk^w^ínem* ranges from 0 = unsuitable to 1 = most suitable. X values are precipitation of the wettest month, in millimeters, from WorldClim contemporary data (1970–2000). Relative habitat suitability was predicted for precipitation of the wettest month while other variables in the WorldClim model were held constant at their mean relative habitat suitability. Values from Skeetchestn Territory are shown with the dark shaded area for the WorldClim present dataset, and the light shaded area for the WorldClim future dataset.

**FIGURE 9:**
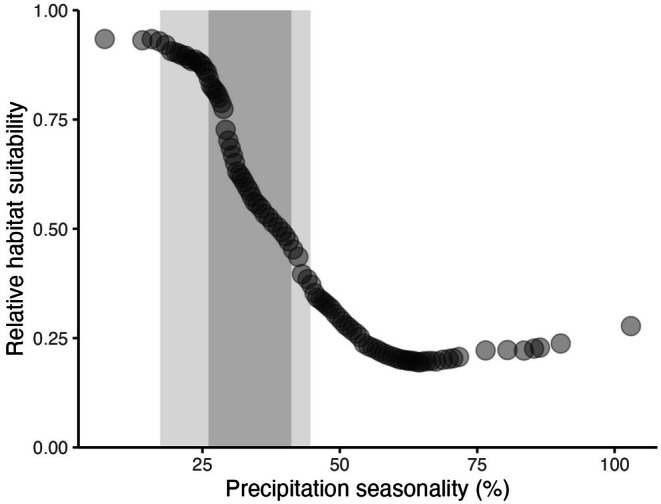
Marginal response curve, based on the WorldClim species distribution model, between bio15 (precipitation seasonality, coefficient of variation) and predicted habitat suitability of *sk^w^enk^w^ínem* ( *Claytonia lanceolata* ). Relative habitat suitability for *sk^w^enk^w^ínem* is predicted to decrease with increasing precipitation seasonality (%). Relative habitat suitability for *sk^w^enk^w^ínem* ranges from 0 = unsuitable to 1 = most suitable. X‐axis values are precipitation seasonality (%) from WorldClim contemporary data (1970–2000). Relative habitat suitability was predicted for precipitation seasonality while other variables in the WorldClim model were held constant at their mean relative habitat suitability. Values from Skeetchestn Territory are shown with the dark shaded area for the WorldClim present dataset, and the light shaded area for the WorldClim future dataset.

The two most influential predictors for the WorldClim model were precipitation of the wettest month (Bio13; Figure 7) and precipitation seasonality (measured as the coefficient of variation for precipitation, Bio 15; Figure 7). Relative habitat suitability for *sk* ^*w*^ *enk* ^*w*^ *ínem* increased with increasing precipitation of the wettest month, up to 150 mm (Figure 8) and decreased with increasing precipitation seasonality (Figure 9).

### 3.3 Predicted Area

**FIGURE 10:**
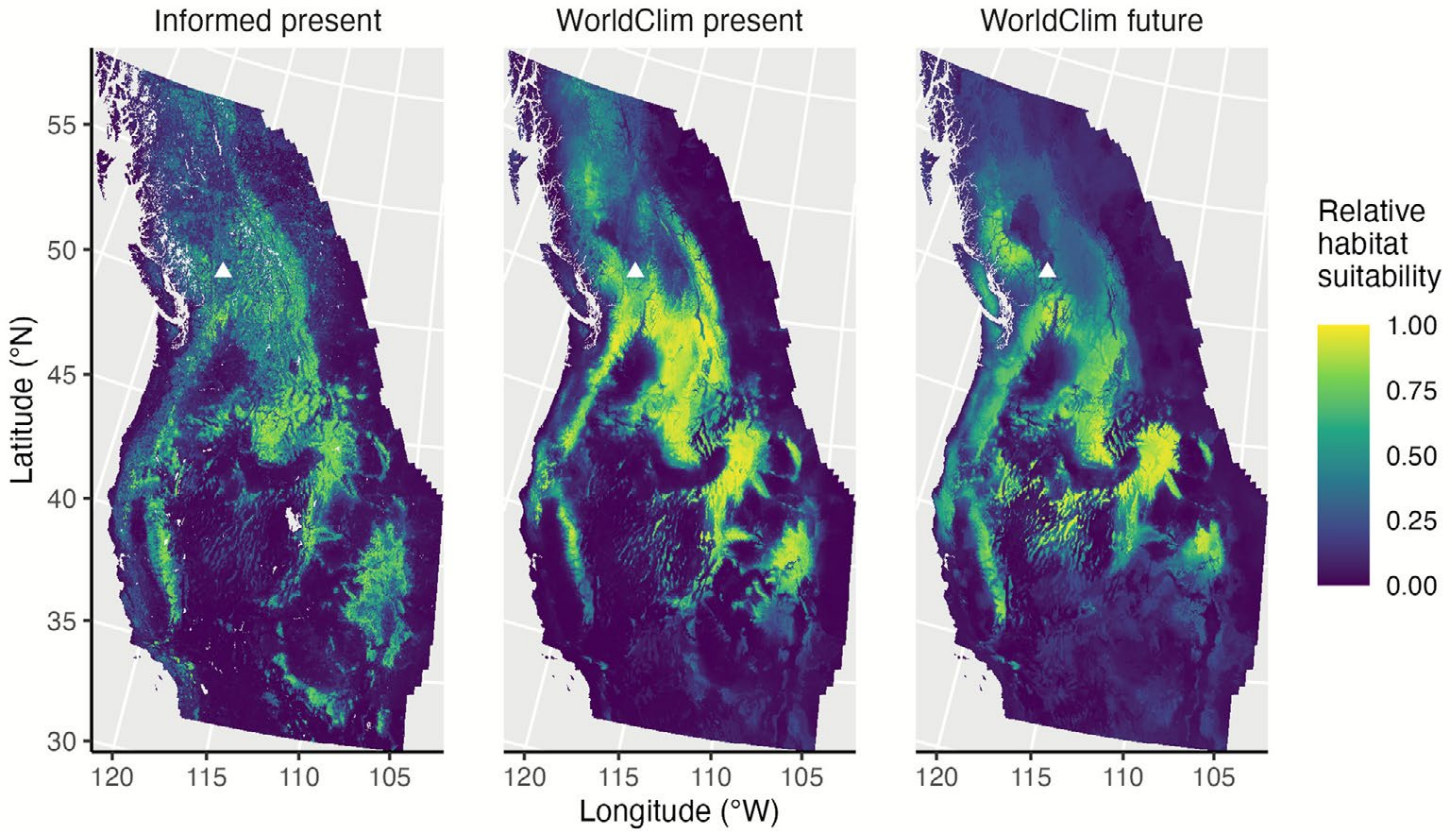
Continuous habitat suitability present and future predictions for *sk^w^enk^w^ínem* ( *Claytonia lanceolata* ) in western North America based on the Informed and WorldClim ensemble species distribution models. The index is relative habitat suitability, ranging from 0 to 1. Predictions are mean average predictions based on their respective ensemble model. The left and middle panels show habitat suitability predictions for the present, and the right panel shows predictions for 2080–2100 under future climate change scenario SSP 5–8.5. The projection used is the North America Albers equal area conic projection. Triangles indicate the general location of Skeetchestn Territory.

Overall, our Informed and WorldClim models predicted similar distributions in suitable habitat for *sk* ^*w*^ *enk* ^*w*^ *ínem* (Figure 10). For the present time, the Informed model predicted a larger suitable area than the WorldClim present model over our full study extent and within Skeetchestn Territory (Table 4). However, the WorldClim present prediction showed more highly suitable habitat than the Informed prediction (Figure 10). The WorldClim future predictions estimated less suitable habitat for *sk* ^*w*^ *enk* ^*w*^ *ínem* over the full study extent and within Skeetchestn Territory, compared to the WorldClim present prediction (Table 4, Figure 10). Over the full study extent, the Informed (33.43%), WorldClim present (28.08%), and WorldClim future (25.59%) predictions classified about 1/4 to 1/3 of the study extent as suitable habitat for *sk* ^*w*^ *enk* ^*w*^ *ínem* (Table 4). The Informed (75.22%) and WorldClim present (70.36%) predictions classified a majority of Skeetchestn Territory as suitable habitat for *sk* ^*w*^ *enk* ^*w*^ *ínem* (Table 4). The WorldClim future (36.29%) prediction classified only about 1/3 of Skeetchestn Territory as suitable habitat for *sk* ^*w*^ *enk* ^*w*^ *ínem* (Table 4). There was a reduction in suitable habitat between the WorldClim present and future predictions, for both the full study extent (−2.49%) and within Skeetchestn Territory (−19.76%). Relative to the predicted suitable area, we predicted a loss of suitable habitat from present to future for the full study extent (−14.06%) and Skeetchestn Territory (−26.26%).

**TABLE 4:** Absolute area (km^2^) and percent area (%), relative to the study area and Skeetchestn Territory, predicted as suitable for *sk* ^*w*^ *enk* ^*w*^ *ínem* ( *Claytonia lanceolata* ) by the Informed species distribution model (present time), and WorldClim species distribution model (present and future predictions).

Prediction	Total study area		Skeetchestn territory	
	Absolute area (km^2^)	Percent area (%)	Absolute area (km^2^)	Percent area (%)
Informed present	1,304,882.00	33.43	5408.10	75.22
WorldClim present	1,130,061.00	28.08	5058.93	70.36
WorldClim future	1,029,659.00	25.59	2608.93	36.29
Change from WorldClim present to WorldClim future	100,401.9	−2.49	−1421.05	−19.76

*Note:* The present WorldClim model uses predictor data from 1970 to 2000, and the future WorldClim model uses predictor data from under Shared Socioeconomic Pathway (SSP) scenario 5–8.5. The Change from WorldClim Present to WorldClim Future shows an overall loss of suitable habitat with SSP scenario 5–8.5. Our total study area is 4,023,545 km^2^ and Skeetchestn Territory is 7190 km^2^. Values are rounded to 2 decimal places.

**FIGURE 11:**
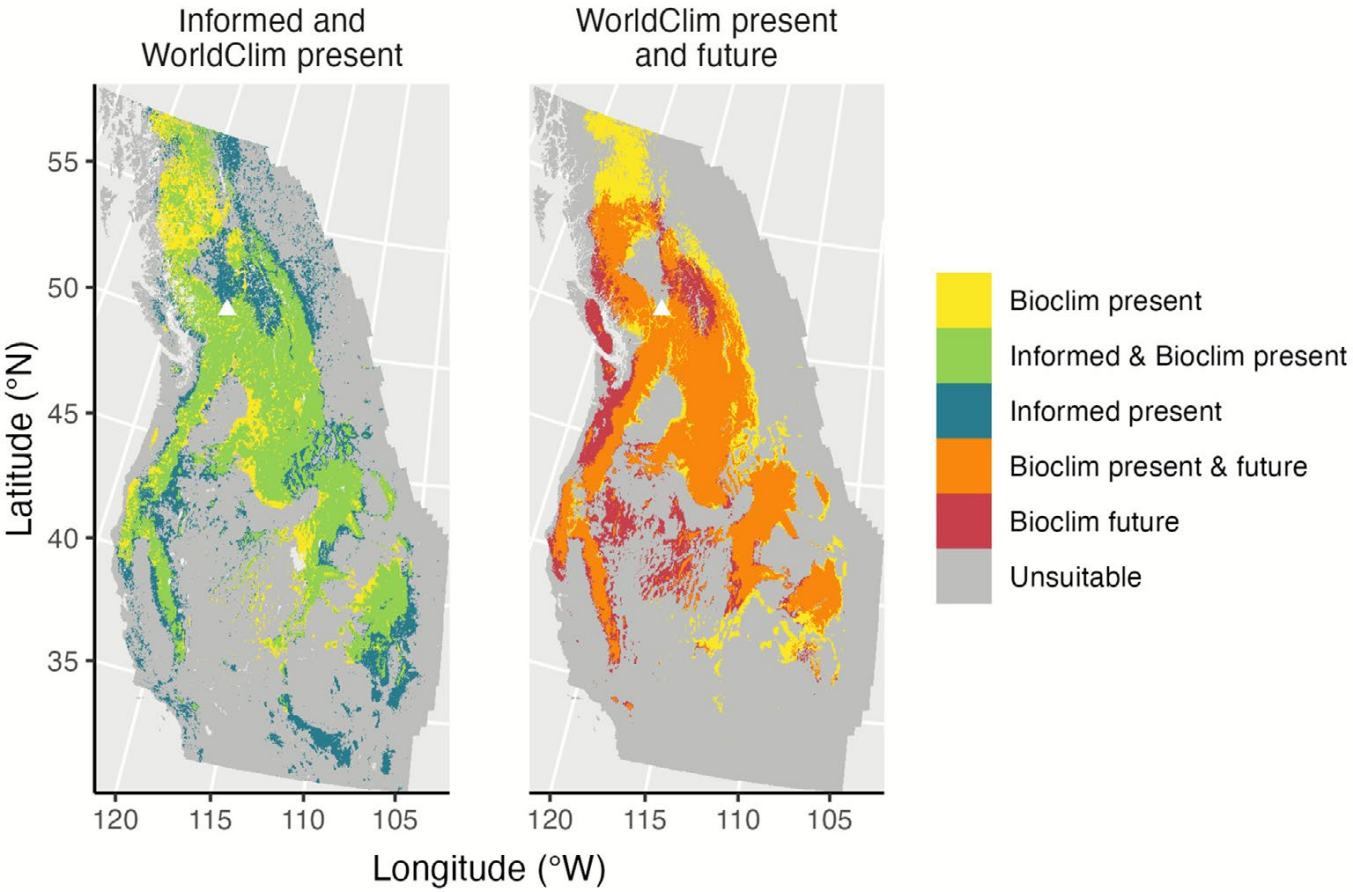
Area of agreement predictions for *sk^w^enk^w^ínem* ( *Claytonia lanceolata* ) based on the Informed and WorldClim species distribution models. Predictions are mean average predictions based on their respective ensemble model, which were thresholded to binary suitable/unsuitable predictions. The left panel shows habitat suitability predictions for the present time, while the right panel shows future predictions. “WorldClim present” is the area predicted to be suitable by the WorldClim model for the present time, and “Informed present” is the area predicted to be suitable by the Informed model for the present time. “Informed & WorldClim” indicates the area predicted to be suitable by both the Informed and WorldClim models. “WorldClim future” is the area predicted to be suitable in 2081–2100 under future climate change scenario SSP 5–8.5, and “WorldClim present & future” is the area predicted to be suitable by both WorldClim present and future predictions. Map projection used is the North America Albers equal area conic projection. Triangles indicate the general location of Skeetchestn Territory.

For the binary suitable habitat predictions, the TSS max threshold (optimum value) for suitable habitat was 0.24 (mean, median, and weighted mean). The area of agreement in suitable habitat between the Informed and WorldClim present‐day predictions represented 21.26% of the full study extent and 56.32% of Skeetchestn Territory (Figure 11). The area of agreement between the WorldClim present and future predictions represented 19.63% of the full study extent (Figure 11) and 36.15% of Skeetchestn Territory. A majority of the total suitable habitat predicted by both the Informed and WorldClim predictions (54.75%) and WorldClim present and future predictions (57.66%) was in agreement over the full study extent. Within Skeetchestn Territory, the overlap between the Informed and WorldClim present predictions represented 63.27% of the predicted suitable habitat, and the overlap between the WorldClim present and future predictions represented 51.28% of the predicted suitable habitat.

## 4 Discussion

Species distribution models are commonly used to inform conservation efforts, but rarely do they include Indigenous input. As Indigenous Peoples are acutely vulnerable to climate change due to their deep connections to and interdependence with the Land (Daigle et al. 2019; Hille 2022), they stand to benefit greatly from SDM studies in which they can provide information. Using Skeetchestn's Knowledge to inform the predictors selected for input into the Informed species distribution model produced ecologically relevant predictions from community participation. We produced continuous habitat suitability indices for *sk* ^*w*^ *enk* ^*w*^ *ínem* over its full distribution, which can be used by Skeetchestn or other Nations interested in *sk* ^*w*^ *enk* ^*w*^ *ínem*'s habitat suitability. Furthermore, the computationally reproducible SDMs produced here can be repeated with other culturally significant plants, with the potential to include different predictors informed by Indigenous Knowledge.

### 4.1 Model Performance

The performances of the Informed and WorldClim models indicated each effectively discriminated between presence and pseudoabsence points during the model cross‐validation (Table 2). The Informed and WorldClim ensemble models displayed negligible differences in model performance with the MaxEnt algorithm, and the WorldClim model had improved performance over the Informed model with random forest (Table 2). Therefore, our chosen set of predictors in the Informed model with MaxEnt can explain the relative suitability of habitat for *sk* ^*w*^ *enk* ^*w*^ *ínem* with the same predictive power as the WorldClim variables, and nearly the same predictive power with random forest (Table 2).

### 4.2 Predictor Contributions

Mean dropout loss represents the effect of iteratively removing a predictor from the model on the model performance. For the Informed and WorldClim models, the maximum mean dropout losses were under 0.28 and 0.15, respectively (Figures 4 & 7). Since mean dropout loss represents 1—AUC loss after permutations, values of predictor importance can be from 0 to 1, with 0 being unimportant and 1 being highly important influencers on model performance (Naimi and Araújo 2016; Zurell 2020). Our maximum mean dropout loss (variable importance) values were closer to 0 than 1, indicating none of the predictors had particularly strong independent influences on the model performance. Another SDM study had similarly low mean relative predictor contributions of 0.471, 0.0762, and 0.0662 for their top three contributing predictors, two of which were soil variables (Ni and Vellend 2022). However, much like the current study, their model performance values are high, with AUC > 0.9 for all three models (Ni and Vellend 2022).

For the Informed model, soil temperature seasonality (°C, 0–5 cm below surface) and elevation (m) were the two most influential predictors on the model's performance (Figure 4), and therefore in classifying suitable habitat for *sk* ^*w*^ *enk* ^*w*^ *ínem*. Within a smaller extent, such as Skeetchestn Territory, elevation is a main driver of species distributions (Pearson and Dawson 2003). Over a larger geographic extent, *sk* ^*w*^ *enk* ^*w*^ *ínem* is widespread and can be considered a relatively generalist plant species, occupying habitat over a large range in elevation (Figure 6). The marginal response plot for elevation indicated the ideal elevation for *sk* ^*w*^ *enk* ^*w*^ *ínem*, over the full study extent, is approximately 2000 m (Figure 6). There is a peak in relative habitat suitability at 2000 m, and the existing literature states *sk* ^*w*^ *enk* ^*w*^ *ínem* is known to grow at elevations ranging from sea level to 3600 m (Davis 1966; Stewart and Wiens 1971; Douglas and Taylor 1972). Our study sites and nearby *sk* ^*w*^ *enk* ^*w*^ *ínem* patches within Skeetchestn Territory are at approximately 1000 m in elevation, which is within the suitable conditions predicted by our Informed model (Figure 6). Within Skeetchestn Territory, elevation ranges from approximately 300–1900 m, encompassing the most highly suitable elevations according to our results (Figure 6).

However, the effect of elevation is indirect, with latitude important to consider along with elevation, and climate being the direct driver of habitat suitability (Austin 2002; Chauvier et al. 2021). Ultimately, climate influences landcover, which in turn influences soil properties, with differing magnitudes of effect at different elevations (Chauvier et al. 2021). Elevation and climate influence human activity, with human activity concentrated at lower elevations (Nogués‐Bravo et al. 2008). Skeetchestn's *sk* ^*w*^ *enk* ^*w*^ *ínem* patches are in montane habitats, at mid‐elevations, with easy access by humans and therefore human disturbances, both positive and negative (Pilat 2024).

Variation in soil temperature as a determinant of habitat suitability is expected for *sk* ^*w*^ *enk* ^*w*^ *ínem*, a geophyte species. *Sk* ^*w*^ *enk* ^*w*^ *ínem* corms are known to grow at shallow depths and require soil temperatures to stay at or above 0°C year‐round, so their corms do not freeze (Miller and Chambers 2006; Briceño et al. 2015). Our results showed high relative habitat suitability at a 6.5°C standard deviation in soil temperature, with a decline in relative habitat suitability above a 7.0°C standard deviation in soil temperature (Figure 5). The higher the standard deviation, the greater the annual variability in soil temperature (O'Donnell and Ignizio 2012). Upon inspection of the soil temperature seasonality raster, high variability in soil temperature occurs in areas such as high elevation deserts where temperatures fluctuate between extreme highs and lows, which were generally predicted to be unsuitable for *sk* ^*w*^ *enk* ^*w*^ *ínem* (Figure 5). The highest habitat suitability was predicted in areas with moderate variability in soil temperature (Figure 5), where snowpack insulates the soil over winter, but soils do not reach temperatures as high as in deserts, for example. Skeetchestn Territory encompasses the conditions in which soil temperatures are highly suitable for *sk* ^*w*^ *enk* ^*w*^ *ínem* (Figure 5). However, soil temperature seasonality is interpolated from air temperature data, which itself is interpolated from numerous weather stations (Karger et al. 2017; van den Hoogen et al. 2021). Therefore, we must be careful when inferring habitat suitability from soil temperature datasets with high uncertainty. Incorporating snowpack or snow persistence predictors into our model could better inform us of soil temperatures over winter, leading to better predictions of habitat suitability for *sk* ^*w*^ *enk* ^*w*^ *ínem*. However, high quality snowpack and snow persistence datasets do not exist over our entire study area, and therefore were not included as predictors in our SDM.

The most influential predictors for the WorldClim model were precipitation of the wettest month (bio13; Figures 7 and 8) and precipitation seasonality/coefficient of variation (bio15; Figures 7 and 9). There was an increase in habitat suitability with increasing precipitation of the wettest month, up to 150 mm where a plateau is reached (Figure 8). Sufficient precipitation as snow, and therefore spring soil moisture, is an important determinant of habitat suitability for *sk* ^*w*^ *enk* ^*w*^ *ínem*, which is hypothesized to emerge following an increase in soil moisture, immediately following snowmelt (Gezon et al. 2016; Jerome et al. 2021). As a geophyte, *sk* ^*w*^ *enk* ^*w*^ *ínem* requires sufficient snowpack to insulate its corms over the winter; otherwise, the reproductive organs freeze and the individual dies (Miller and Chambers 2006; Briceño et al. 2015). We chose not to include temperature seasonality (bio4) or minimum temperature of the coldest month (bio6) in the WorldClim model because not only were they found to be highly correlated with other variables, but we assume that with sufficient snowpack in the winter, air temperature in the coldest months would not have an impact on the temperature of the soil.

For precipitation of the wettest month, *sk* ^*w*^ *enk* ^*w*^ *ínem* likely requires the wettest months to occur over winter, so sufficient precipitation as snow accumulates and their corms are insulated. Precipitation during the months following senescence is not likely to be a strong determining factor in *sk* ^*w*^ *enk* ^*w*^ *ínem* habitat suitability, as the corms have entered dormancy prior to the hot summer months. The use of snow persistence (day of year of average snowmelt) in SDMs has been shown to improve predictions for cold‐adapted species in Fennoscandia, with snow persistence argued as a driver of plant distributions (Rissanen et al. 2021). Although our study system is in lower and warmer latitudes, the phenology of *sk* ^*w*^ *enk* ^*w*^ *ínem* is dependent on the timing of snowmelt, making snow persistence a determining factor in its habitat suitability.

For precipitation seasonality, highly suitable habitat was associated with low variation in precipitation, with a sharp decrease in habitat suitability with higher variability (Figure 9). The inverse relationship between precipitation seasonality and habitat suitability is an unexpected finding, as *sk* ^*w*^ *enk* ^*w*^ *ínem* is thought to be adapted to annual fluctuations in precipitation. However, it is possible many of the *sk* ^*w*^ *enk* ^*w*^ *ínem* occurrences are in areas with similar amounts of precipitation as rain and precipitation as snow, leading to lower variability in annual precipitation but not necessarily less snowpack. Another possible explanation is some populations of locally adapted cytotypes may be suited to habitat with less annual variability. For example, in the Cascade Mountains, snow release occurred throughout June, July, and August, with morphologically taller varieties of *Claytonia* associated with earlier snowmelt, and shorter varieties of *Claytonia* associated with later snowmelt (Douglas and Taylor 1972). The authors suggest belowground plant development occurred in the same way whether there was 2 cm or 2 m of snow over winter (Douglas and Taylor 1972). These findings show annual variability in precipitation might not be as important as initially thought for determining suitable habitat, with the possibility of several different cytotypes adapted to many different conditions over the large extent in which *sk* ^*w*^ *enk* ^*w*^ *ínem* exists.

Landcover was the least influential predictor in our Informed model (Figure 4). Based on the Knowledge of *sk* ^*w*^ *enk* ^*w*^ *ínem* within Skeetchestn Territory (Pilat 2024), *sk* ^*w*^ *enk* ^*w*^ *ínem* is associated with Douglas fir (*Pseudostuga menziesii*) forest openings, so we expected landcover to be a strong predictor of suitable habitat. However, landcover was a less influential predictor than climate for the distribution of 2616 plants in the European Alps (Chauvier et al. 2021). The influence of landcover is tied to soil properties and climate, with the individual effects of landcover alone difficult to disentangle (Chauvier et al. 2021). The landcover raster used in our analysis had a spatial resolution of 30 m, which was then upscaled to 30 arcseconds (1 × 1 km at the equator) resolution to match our other predictors. However, the forest openings in Skeetchestn Territory are often < 10 m^2^ (Pilat 2024), so the resolution of this dataset does not adequately capture this habitat type. We recommend using higher resolution landcover data to investigate if the forest openings in which *sk* ^*w*^ *enk* ^*w*^ *ínem* grows will be captured in the model predictions, and to improve model performance and predictive accuracy (Chauvier et al. 2022).

Anthropogenic biomes were the second least influential predictor in the Informed model (Figure 4). Coupled with landcover, anthropogenic biomes characterize habitat fragmentation and human influence on ecosystems (Ellis and Ramankutty 2008; Chauvier et al. 2021), which are prevalent in Skeetchestn Territory. The anthropogenic biomes dataset was available at a coarser resolution (5 arcminute) and was downscaled to 30 arcsecond resolution. We recommend using higher resolution land‐use data to better capture the small‐scale land uses within our study extent. It is also important to consider that species may not be at equilibrium in their environment. In Skeetchestn Territory, *sk* ^*w*^ *enk* ^*w*^ *ínem* tends to be found in meadows where cattle graze, and is known to be declining in quality (Pilat 2024). This is the disadvantage of using correlative species distribution models; although the species may exist in a certain area, the species may be declining in quality, and an SDM will not capture this relationship. Indigenous Knowledge of the quality of focal species is important to consider in parallel with SDM results, such as our complementary qualitative study (Pilat 2024), under the framework of Two‐Eyed Seeing.

### 4.3 Predicted Area

The areas predicted to be suitable by the Informed and WorldClim present models comprised approximately 30% of our total study area, while the WorldClim future prediction comprised approximately 25% of our total study area (Table 4, Figures 10 and 11). This was a considerable portion of the large extent under study. Out of the total suitable area predicted within our full study extent, the Informed and WorldClim present predictions had a majority overlap (Figure 11). Because the present‐day predictions are similar between the Informed and WorldClim present models, we assume the WorldClim model with its selected predictors can be a useful visualization for where suitable habitat might exist under a possible future scenario.

Conservation efforts should target *sk* ^*w*^ *enk* ^*w*^ *ínem* patches within the area of agreement from the WorldClim present and future predictions, as habitat contained within the overlap is likely to be highly suitable for *sk* ^*w*^ *enk* ^*w*^ *ínem* now and for decades to come. In the context of Skeetchestn Territory, the Informed and WorldClim present predictions had approximately 1/2 of their predicted suitable area in agreement, while the WorldClim present and future predictions had around 1/3 overlap in predicted suitable area. We predicted approximately 1/4 of Skeetchestn Territory classified as suitable in the WorldClim present prediction to become unsuitable in the future, while 14% of the total habitat classified as suitable in the WorldClim present prediction is predicted to become unsuitable in the future. An increase in suitable habitat over the Territory indicates new areas of Skeetchestn Territory are predicted to become suitable, while some of the currently suitable habitat is predicted to become unsuitable.

Although there was a reduction in absolute area predicted to be suitable over the full study extent, much of the suitable habitat was predicted to remain suitable in the future, which is an ideal scenario (Figures 10 and 11). Visually, the northernmost suitable habitat in the WorldClim present prediction is not considered suitable in the future (Figures 10 and 11). Habitat classified as highly suitable changed slightly in the future (Figure 10). A common trend for species distribution shifts with climate change is poleward shifts, so our findings that the northern habitats may become unsuitable are unexpected (Parmesan and Yohe 2003; Parmesan 2006; Dyderski et al. 2018; Chakraborty et al. 2021). However, a study investigating the effect of soil predictors on plant species distribution models found the inclusion of soil predictors, rather than climate predictors alone, predicted smaller magnitudes of latitudinal shifts in distribution with climate change (Ni and Vellend 2022). Our future prediction showed a retraction in distribution from the northern portion of our study area with climate predictors alone, indicating that the distribution of *sk* ^*w*^ *enk* ^*w*^ *ínem* may retract to the central portion of our study area. Including future soil variables in a model transfer may further support this finding if both high latitude soils and climate are not suitable for *sk* ^*w*^ *enk* ^*w*^ *ínem*.

Our models predicted a decrease in suitable habitat in Skeetchestn Territory in the future, and no areas in Skeetchestn Territory showed particularly high habitat suitability for *sk* ^*w*^ *enk* ^*w*^ *ínem*. However, upon visually inspecting the continuous habitat suitability maps of Skeetchestn, some areas were predicted to have higher relative habitat suitability in the future than the present predictions from the WorldClim model. The Informed model shows low habitat suitability for *sk* ^*w*^ *enk* ^*w*^ *ínem* within Skeetchestn Territory. Comparing the Informed present prediction to a future prediction with the same predictor variables could give better insight into how projected anthropogenic effects (such as land‐use changes) may impact suitable habitat for *sk* ^*w*^ *enk* ^*w*^ *ínem*. Although the climate may become more favorable based on temperature and precipitation, the impacts of snow persistence with climate change were not tested in this analysis. Since snowmelt timing is thought to be a limiting factor in *sk* ^*w*^ *enk* ^*w*^ *ínem*'s success, we strongly recommend including snow persistence as a predictor in future analyses, once high‐quality datasets become available. We also recommend using Territory‐specific occurrence records to better characterize suitable habitat in Skeetchestn Territory. We emphasize our prediction represents one possible scenario, and *sk* ^*w*^ *enk* ^*w*^ *ínem* may respond to climate change and anthropogenic factors in unexpected ways.

### 4.4 Limitations

The use of presence‐only data with pseudoabsence points means we cannot infer the probability of presence; we can only estimate relative habitat suitability (Lee‐Yaw et al. 2022; Soley‐Guardia et al. 2024). We also cannot infer the causes of the distribution of *sk* ^*w*^ *enk* ^*w*^ *ínem*, since SDMs are correlative (Guisan and Zimmermann 2000; Austin 2002). What we can infer are relationships between environmental predictors and *sk* ^*w*^ *enk* ^*w*^ *ínem* habitat to estimate which conditions are suitable. Although this study predicts potential habitat suitability for *sk* ^*w*^ *enk* ^*w*^ *ínem* into the future, we could only do so with bioclimatic variables from WorldClim, as the variables used in our Informed model are not available for the future. This means our future projection did not consider anthropogenic influences, soil conditions, or snow persistence, which are thought to be limiting to the distribution of geophytes like *sk* ^*w*^ *enk* ^*w*^ *ínem* in space (Chauvier et al. 2021; Ni and Vellend 2022). Interpreting the future predictions of suitable habitat for *sk* ^*w*^ *enk* ^*w*^ *ínem* should be approached with caution, as anthropogenic influences will change between now and 2081–2100. We recommend subsequent studies incorporate future predictions based on anthropogenic and land use variables, when available, to better inform the potential distribution of *sk* ^*w*^ *enk* ^*w*^ *ínem*.

Our model produced landscape‐level predictions, based on the known realized niche of *sk* ^*w*^ *enk* ^*w*^ *ínem*. We encountered computation limits when using predictors at 30 arcsecond resolution. These 30 arcsecond projections are visually informative on a large scale, but cannot capture the small *sk* ^*w*^ *enk* ^*w*^ *ínem* patches known to exist in Skeetchestn Territory. We recommend using higher resolution data for finer‐grain predictions to better capture the microhabitats in which *sk* ^*w*^ *enk* ^*w*^ *ínem* patches tend to be found, ultimately producing predictions that could be more relevant to individual communities (McBride et al. 2017). However, using lower resolution data means we can produce publishable habitat suitability indices without compromising specific resource locations. The future predictions illustrate if the areas of high cultural importance where *sk* ^*w*^ *enk* ^*w*^ *ínem* is known to grow today were predicted to become unsuitable in the future. These results can inform a targeted approach to *sk* ^*w*^ *enk* ^*w*^ *ínem* patch conservation, to account for the larger climatic changes beyond the control of the community. Although future insights are useful, we need to be cautious when projecting models to novel temporal conditions (Crimmins et al. 2013; Moreno‐Amat et al. 2015; Feng et al. 2019; Low et al. 2020). Ensemble models, including those using random forest, evaluated with temporally independent data had lower performance than models evaluated with spatially independent data (Crimmins et al. 2013). A similar phenomenon exists for Maxent where model performance decreased when transferred to novel environments (Feng et al. 2019). We recommend future habitat planning studies quantify the uncertainty of model transfers to guide the interpretation of the predictions (Moreno‐Amat et al. 2015; Feng et al. 2019; Low et al. 2020).

### 4.5 Two‐Eyed Seeing Approach for SDMs

The species distribution model literature is vast but severely lacking in studies that partner with Indigenous communities to inform their modeling approach and predictor variable selection (but see Doyle‐Yamaguchi 2021; Skroblin et al. 2021; Campbell et al. 2022; Mucioki et al. 2022). Furthermore, SDMs that are informed by Indigenous Knowledge, aligned with the principles of OCAP (First Nations Information Governance Centre 2024), and computationally reproducible are rare, if they exist at all (Pilat 2024). Given that Indigenous Peoples are particularly vulnerable to climate change (Daigle et al. 2019; Hille 2022), their participation in SDM studies is of high importance for producing powerful predictions and relevant results. Reproducible SDM studies are also of critical importance for the broader applicability of the study. Following a Two‐Eyed Seeing framework, Indigenous Knowledges can better inform predictor selection, as opposed to using the standard bioclimatic variables, which may not be particularly relevant to the species under study.

Our study provides a baseline estimate and future prediction of suitable habitat for *sk* ^*w*^ *enk* ^*w*^ *ínem* over its known distribution, so Indigenous communities other than Skeetchestn may also find our results useful for revitalizing their *sk* ^*w*^ *enk* ^*w*^ *ínem* patches. Publishing the maps illustrating the full distribution of *sk* ^*w*^ *enk* ^*w*^ *ínem* maintains the broader utility of this study but keeps the Skeetchestn‐specific maps in the hands of Skeetchestn. Following a Two‐Eyed Seeing approach, the SDM process should be repeated with the inclusion of Territory‐specific occurrence data to better incorporate Indigenous Knowledge in the model inputs. We recommend future studies also co‐interpret the results of SDM studies with Indigenous communities to better analyze the trustworthiness of present‐day predictions. Indigenous Peoples know their Lands best, so a joint effort to both inform and interpret SDMs would be an ideal Two‐Eyed Seeing approach. Future research should follow the lead of Indigenous communities to produce useful tools while respecting their rights to stewardship and data sovereignty, following the principles of OCAP (First Nations Information Governance Centre 2024).

## 5 Conclusion

Our Informed and WorldClim models had high predictive performances and similar predictions of relative habitat suitability in space over the full extent of our study area. Within Skeetchestn Territory, these predictions were different in terms of area predicted to be suitable, with more relative area in agreement than over the full study extent. Our future predictions showed a decrease in suitable habitat from the present to 2081–2100, under the most pessimistic climate change scenario, over the full study extent and within Skeetchestn Territory. Territory‐specific data will likely result in a different future prediction, which could give better insight into the future suitable habitat available to *sk* ^*w*^ *enk* ^*w*^ *ínem* within Skeetchestn Territory.

While species distribution modeling through a Two‐Eyed Seeing approach is a useful framework, there are gaps to be filled and many considerations to take in this approach. The balance of using high‐resolution data and having computational limits is important when conducting SDM studies for Indigenous communities who have localized interests. A deep understanding of the broader ecological system, such as that provided by Indigenous Knowledge, is critical to making informed predictor selections and analyzing the predictions from SDMs, following a Two‐Eyed Seeing approach. Although there are shortcomings to using correlative statistical models, the resultant maps serve as visualizations of cultural use and baseline estimates of habitat suitability for future monitoring efforts, with the potential to support Skeetchestn in exercising sovereignty over their *tmicw* (lands, waters, and all living creatures).

## Author Contributions

**Hannah E. Pilat:** conceptualization (equal), data curation (equal), formal analysis (lead), funding acquisition (equal), investigation (lead), methodology (lead), project administration (lead), resources (equal), visualization (lead), writing – original draft (lead), writing – review and editing (equal). **David J. Ensing:** conceptualization (equal), data curation (equal), formal analysis (supporting), funding acquisition (equal), investigation (equal), methodology (equal), project administration (equal), resources (equal), supervision (equal), visualization (supporting), writing – original draft (supporting), writing – review and editing (equal). **Jason Pither:** conceptualization (equal), data curation (equal), formal analysis (supporting), funding acquisition (supporting), investigation (equal), methodology (equal), project administration (equal), resources (equal), supervision (equal), visualization (supporting), writing – original draft (supporting), writing – review and editing (equal).

## Conflicts of Interest

The authors declare no conflicts of interest.

## Data Availability

Data sources and the ODMAP protocol are all included in the study's pre‐registration, available at https://doi.org/10.17605/OSF.IO/RM9S2. Publicly available code is available at https://github.com/hpilat/skwenkwinem_sdm.
